# Public Information Influences Sperm Transfer to Females in Sailfin Molly Males

**DOI:** 10.1371/journal.pone.0053865

**Published:** 2013-01-16

**Authors:** Sabine Nöbel, Klaudia Witte

**Affiliations:** Section of Biology, Research Group of Ecology and Behavioral Biology, University of Siegen, Siegen, Germany; CNRS, Université de Bourgogne, France

## Abstract

In animals, including humans, the social environment can serve as a public information network in which individuals can gather public information about the quality of potential mates by observing conspecifics during sexual interactions. The observing individual itself is also a part of this information network. When recognized by the observed conspecifics as an audience, his/her presence could influence the sexual interaction between those individuals, because the observer might be considered as a potential mate or competitor. One of the most challenging questions in sexual selection to date is how the use of public information in the context of mate choice is linked to the fitness of individuals. Here, we could show that public information influences mate-choice behaviour in sailfin molly males, *Poecilia latipinna,* and influences the amount of sperm males transfer to a female partner. In the presence of an audience male, males spent less time with the previously preferred, larger of two females and significantly more time with the previously non-preferred, smaller female. When males could physically interact with a female and were faced with an audience male, three audience females or no audience, males transferred significantly more sperm to a female partner in the presence of an audience male than with female audience or no audience and spent less time courting his female partner. This is the first study showing that public information use turns into fitness investment, which is the crucial factor to understand the role of public information in the dynamic processes in sexual selection.

## Introduction

Theories of sexual selection assume that female and male mate preferences are genetically determined (overview in [1]–[7]). This has been shown in an enormous number of experimental studies throughout the animal kingdom, including invertebrates and vertebrates [8]–[11]. In most species, females are the choosy sex, because females invest more time, resources and energy in offspring than males [12]. However, males should be choosy as well when they invest energy and time in offspring (like large spermatheca, investment in brood care, etc.) and/or when choosy males have a higher reproductive output than males which paired randomly with females [12]. Even in promiscuous systems, it is adaptive for males not to copulate with any female of the population but instead copulate with high quality females [13].

Although female and male mate preferences are genetically determined, both sexes in a wide range of taxa are able to use public information to evaluate prospective mates [14]–[18]. An individual can extract specific information about a prospective mate (e.g. fighting ability, fighting tactics, body condition, attractiveness, willingness to cooperate, etc.) by interacting directly with this conspecific or indirectly by observing this individual when interacting with others, i.e. by eavesdropping on that individual [19]–[22].

Eavesdropping is defined as the act of extracting information from signalling interactions between conspecifics [20] and occurs when information from an individual transmitting a signal to another individual is picked up by one or more bystanders towards whom the signal was not directed [22]. Eavesdropping is a widespread phenomenon and occurs in many different species like mammals (e.g. [23]–[25]), birds (e.g. [26]–[30]), amphibians [31], fish (e.g. [32]–[35]) and even in insects [36]. The information an eavesdropper can gain is not always reliable, because one or both of the two interacting individuals might be cheating each other during interaction dependent on the presence or absence of an eavesdropper [37]. However, the probability that the eavesdropper gathers reliable information about those individuals is high when he/she is not recognized by the interacting individuals.

In the context of mate choice, eavesdropping can be an effective way for males and females to evaluate potential mates by observing two individuals during sexual interaction. Eavesdroppers can avoid some costs of mate sampling and mate choice by gaining information about mate quality without being directly involved in an interaction with conspecifics. These costs are for example sampling time, risk of sexual harassment, risk of predation or infection (e.g. [4], [38]–[40]). Moreover, eavesdroppers may be able to watch interactions between several conspecifics at the same time, which allows direct comparisons. Additionally, eavesdroppers gain information on the relative quality of mates at little cost and/or risk [21].

One form of eavesdropping in the context of mate choice is mate-choice copying. So far, mate-choice copying is defined only in the context of heterosexual mate choice. Mate-choice copying occurs when an eavesdropper is observing a sexual interaction between two heterosexual conspecifics. The eavesdropper might then copy the mate choice of others by preferring the same individual as a mate [41]–[45], or rejecting the same individual, as the interacting individual did before [46]. Gibson et al. [47] suggested that individuals could benefit from copying when mate assessment is difficult. Nordell and Valone [43] predicted that individuals with poor discrimination ability should copy more often than individuals with superior discrimination ability. Copying increases the probability to mate with a higher quality mate than choosing by chance and is still beneficial when copying a ‘wrong’ choice [43]. Mate-choice copying can have a strong impact on the variance in reproductive success in individuals [47]–[51]. In principle, mate-choice copying can have dramatic effects on the distribution of male traits in a population by altering the distribution of genes passed on to the next generation [50], [52]. Several theoretical models have investigated how mate-choice copying can evolve and be maintained in a population [42]–[44], [51]–[57].

The first experimental evidence of mate-choice copying was found in the guppy *Poecilia reticulata* [41]. Since then, mate-choice copying has been experimentally demonstrated in several bird species, such as the black grouse *Lyrurus tetrix* [58], the greater sage grouse *Centrocercus urophasianus* [47] and the Japanese quail *Coturnix japonica* [59]–[61] and in mammals, such as the Norway rat *Rattus norvegicus* or humans [62]–[64] and even in *Drosophila melanogaster* ([65], [66], but not in *D. serrata* [67]). Up to now, most studies on mate-choice copying have been done in several fish species (e.g. in the guppy *P. reticulata* [68]–[73], in the sailfin molly *Poecilia latipinna* [45], [46], [74]–[80], in the Amazon molly *Poecilia formosa* [81], in the pipefish *Syngnathus typhle* [82], in the stickleback *Gasterosteus aculeatus* [83], in the white belly damselfish *Amblyglyphidodon leucogaster* [84], in the ocellated wrasse *Symphodus ocellatus* [85]) and it has been demonstrated in wild fish populations as well [78], [86].

Mate-choice copying has been experimentally shown in females but also in males in the same species (*P. latipinna* [75], [78], *P. mexicana* [81], [87]). Because only a small proportion of female mollies is receptive at a specific time, males may benefit by copying the choice of other males, i.e. by observing other males in mate choice and see whether a female is receptive or not. To test whether a female is receptive a male nips at her genital opening [88]. Male mate-choice copying can therefore lower the cost of searching for a mate.

The eavesdropper, however, does not only gain information about the two interacting individuals, but the presence of the eavesdropper, when he/she is recognized by others, may influence the nature of the interaction between the observed individuals, which is called the ‘audience effect’ [89]. This so-called ‘audience effect’ or ‘bystander effect’ has been investigated intensively in several species [90]–[93] and especially in fish species (e.g. Siamese fighting fish *Betta splendens* [34], [35], *G. aculeatus* [94]). In Poeciliids, the audience effect has been investigated in *P. mexicana* [95]–[98], *P. reticulata* [99], *P. latipinna* [100] and *Xiphophorus birchmanni* [101]. Plath et al. [96], [97] tested *P. mexicana* males for their mate preferences and found that males changed their initial preference for larger females in presence of a conspecific audience male and spent more time with smaller females. Without an audience, Atlantic molly males chose consistently and preferred larger over smaller females. Plath et al. [97] interpreted these results in the manner that focal males recognized the audience male as a competitor and tried to deceive the audience male about their real mating preference to avoid sperm competition, because surrounding males may use public information and copy the focal males’ mate choice. Thus, sperm competition might increase for focal males. Ziege et al. [98] could show that focal males showed a weaker expression of mating preference when being observed by a rival. This suggests that focal males tried to fake their mating preference to prevent surrounding males from copying their mate choice.

In contrast to a male audience, a female audience might be recognized by a male interacting with a female as a potential mate, which might copy the mate choice of the other female. Thus, public information has an important impact on male mate-choice behaviour.

The most important question, however, is how the use of public information is linked to fitness. One mechanism might be that males adjust sperm transfer to a female partner according to public information, i.e. to the specific type of audience. Sperm competition theory predicts that males should be able to adjust sperm production to the number of rival males and thus, to the level of sperm competition risk and sperm competition intensity. Several experimental studies in a wide range of taxa including humans [102]–[105] have shown that males are able to adjust sperm production in response to different levels of sperm competition. In Poeciliids, males can control both, sperm production and sperm transfer. Guppy males (*P. reticulata*) produce more sperm under low predation risk and it was assumed that sperm competition is more intense when predation pressure is relaxed [106]. Males of the eastern mosquitofish (*Gambusia holbrooki*) raised under high sperm competition conditions (2 males with 3 females) courted more and transferred more sperm to a female in a mating trial than males raised under low sperm competition conditions (1 male with 4 females) [103]. When Atlantic molly males (*P. mexicana*) had the opportunity to mate with two conspecific females and two heterospecific Amazon molly females (*P. formosa*), males preferred to mate with conspecific females and transferred less sperm during copulation with heterospecific Amazon molly females [107]. This is a very precise, controlled and economical mechanism of sperm transfer in *P. mexicana* males and a fascinating adaptation to a socio-sexual situation of the Atlantic molly who lives in sympatry with Amazon mollies, a gynogenetic hybrid species originating from hybridization events between *P. latipinna* and *P. mexicana* [108]. Male sailfin mollies (*P. latipinna*) produce more sperm within one week when they have visual and olfactory contact to conspecific females than with visual and olfactory contact to Amazon molly females [109], [110]. In other species, it has been shown that the presence of rival males is an important cue that males use to fine-tune their mating behaviour in response to the perceived risk and intensity of sperm competition [111]–[115]. Thus, according to sperm competition theory and public information use in mate choice, a male should transfer more sperm to the female partner in the presence of a male audience than without an audience. In the case of a female audience, males should save sperm and transfer less to the female partner to be able to copulate with and transfer sperm to audience females immediately.

Here, we tested, first, whether sailfin molly males (*P. latipinna*) change their mate-choice behaviour in the presence of an audience male in a binary choice situation (audience experiment), and, second, whether males adjust sperm transfer to a female partner according to public information (male audience, female audience, no audience; sperm transfer experiment) in a no-choice situation. We chose a no-choice situation to be able to measure the exact amount of sperm transferred during copulation without possible confounding effects due to a second male.

The sailfin molly is a good model species to investigate a link between the use of public information and fitness measured as the amount of sperm transferred to a female. Sailfin molly males copy each otherś mate choice [75], [78] and males are able to adjust sperm transfer to different situations [109], [110].

## Materials and Methods

### Ethical Statement

Individuals of *P. latipinna* from the Comal River population were caught with dip nets in 2007 (Texas Parks & Wildlife Fishing License No. 512-3894820) and individuals from the Coleto Creek population were caught with dip nets by a commercial fish seller (Goliad Farms, Goliad, Victoria, USA) in 1998 and kept there in natural ponds. These fish were transported in oxygen-enriched plastic bags inside styrofoam boxes as air cargo via Goliad Farms, Goliad, Victoria, USA (Export References Protocol 755-1208, Import Permit V3-19J 06.07), to our lab in January 2009. All behavioural experiments were performed in the Research Group of Ecology and Behavioral Biology, Section of Biology, under the permission of the County Veterinary Office, Siegen, Germany (Permit No. 53.6 55-05).

We declare that this study was carried out in strict accordance with the recommendations in the Guide for the Care and Use of Laboratory Animals of the German Right of Animal Welfare (Tierschutzgesetz). After the German Right of Animal Welfare (Tierschutzgesetz) no authorization shall be required for planned experiments taking the form of vaccinations, withdrawal of blood samples or any other diagnostic measures in line with proven methods **(Article 8 (7.2) Animal Welfare Act)**. We anaesthetised the fish with MS 222 to sample spermatozoa. This is a diagnostic measure in line with a proven method. We notified the person for Animal Welfare at the University of Siegen, Dr. Thomas Hoppe, and we notified the committee for Animal Welfare at the State Office Landesamt für Natur, Umwelt und Verbraucherschutz Nordrhein-Westfalen, the approving authority for experiments with animals.

### Study Species

Sailfin mollies are live-bearing poeciliid fish without parental care. They live in mixed-sex shoals comprising 10 to 20 individuals. Females and males have the opportunity to observe other conspecifics during mate choice and copy the choice of other females and males [78]. All fish used in the audience experiment were mature descendants of wild fish from the Coleto Creek near Victoria, Texas, USA, caught in 1998 and the fish used in the sperm transfer experiment were at least 6 months old and descendants of wild fish from the Comal River near New Braunfels, Texas, USA, caught in summer 2007.

All fish used in the experiments were housed in mixed-sex shoals, each with approximately 50 individuals, in three large stock tanks (98 cm×48 cm×39 cm) with a constant temperature (22−25°C) and a light-dark-cycle of 14∶10 hours. They were fed daily with JBL flake food, frozen Chironomidae larvae or frozen *Artemia* spec. One week before experiments started fish were separated by sex and sheltered under the same light and food regime in three large tanks (each 98 cm×48 cm×39 cm), two for the females and one for the males. During testing period, test fish were separated in single tanks (40 cm×25 cm×41 cm) kept under the same light regime and nutrition condition as in stock tanks. After experiments all fish were released back into their stock tanks.

### Audience Experiment

**Figure 1:**
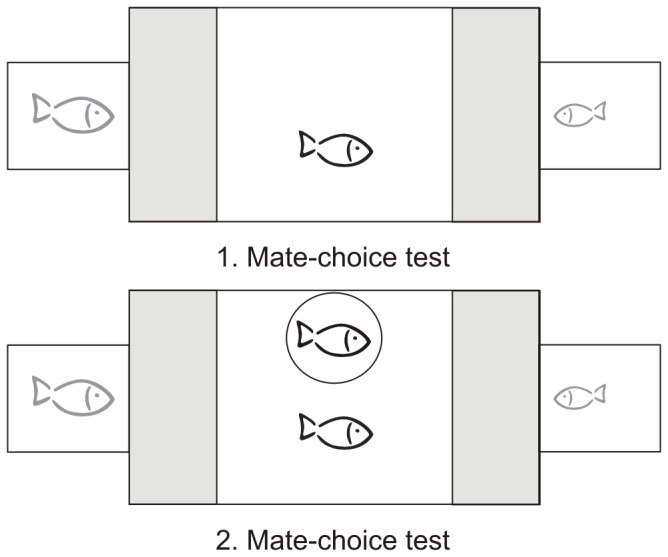
Top view on the experimental set-up of the audience experiment. We used a test tank for the focal male and two tanks for stimulus females. Males (black fishes) could choose between a large and a small female (grey fishes). We measured the time the focal male spent within the mate-choice zones (grey areas) in front of each female. In the second part of the mate-choice test, the focal male chose between the same females in the presence of an audience male inside a clear Plexiglas cylinder. Focal males and audience males were matched for body length. Stimulus females differed in at least 5 mm in body length.

Experiments were performed in a large test tank (100 cm×50 cm×40 cm) and a small stimulus tank (20 cm×25 cm×40 cm) adjacent to each end of the large tank (see Fig. 1). Each tank had gravel on the ground and was filled with water up to 25 cm. Water temperature was 22±1°C. The backsides of the tanks were covered with blue plastic foil to avoid any disturbances from outside during experiments. Two fluorescent tubes (2×60 W) were placed centrally 100 cm above the set-up. Before and after, but not during experiments, water was aerated by a filter and additionally by an air stone. We marked a mate-choice zone in the large test tank 20 cm in front of each stimulus tank with black vertical bars on the front glass and two clear glass sticks laying on the gravel 20 cm apart from both ends of the test tank.

First, the focal male was placed into the test tank and two stimulus females (different in body length of at least 5 mm) were placed into the stimulus tanks at each side of the test tank. Opaque screens were inserted between tanks. After 20 min, the focal male was gently placed into a clear Plexiglas cylinder (11 cm diameter) in the middle of the large tank, and the opaque screens were removed. During this period, the focal male was able to observe both stimulus females for 10 min. After the acclimatisation period, we released the male out of the cylinder and recorded the time he spent within the 20 cm mate-choice zone in front of each stimulus tank for 10 min. We then reinserted the opaque screens, placed the male back into the Plexiglas cylinder and switched the females between the two tanks and repeated the mate-choice trial. The focal male was considered to prefer a particular stimulus female if he spent more time within the mate-choice zone in front of that particular female during the two 10-min mate-choice trials.

A focal male was considered to be side biased if he spent more than 90% of the total time in the same mate-choice zone in both 10-min trials although females had been switched. Those males were rejected from the analysis.

After this first mate-choice test, we placed the focal male back into the Plexiglas cylinder for 2 min and placed a conspecific male (of similar body length as the focal male) as an audience in another Plexiglas cylinder (11 cm diameter) next to the focal male. Then, we released the focal male into the tank and recorded the time he spent within the mate-choice zones in front of the same two females for two 10-min mate-choice trials with switching the females after the first 10-min trial.

Each focal male was used only once per experimental set as a focal male, but it was used as an audience male in another test after a break of at least 5 days. After each test, we measured body size as the standard body length from the tip of the snout to the end of the caudal peduncle in males and females. Each stimulus female was used in two different tests, but in the second test paired with a different female stimulus.

We tested 14 focal males. Two males showed side biases and were removed from the analysis. Focal males (*N* = 12) and the audience males (*N* = 12) were matched for body length. Focal males had an average (± SD) body length of 42.3±6.8 mm and audience males of 43.0±6.1 mm. The stimulus females in each test differed in standard body length in at least 5 mm. The smaller females had an average body length of 28.0±1.0 mm and larger females of 46.6±3.9 mm.

### Control for the Audience Experiment

The control was performed in the same set-up under same condition and the same procedure as the experiment. The only difference was that the second Plexiglas cylinder in the second mate-choice test contained no audience male.

We tested 14 focal males with 14 pairs of females. The average (± SD) body length of focal males (*N* = 14) was 44.7±5.7 mm. Females differed in body length more than 5 mm in each test (smaller females 28.0±4.1 mm, larger females 41.8±5.8 mm, one data point missing).

### Sperm Transfer Experiment

In this experiment, we either presented a single male audience, three fertile females, or no audience to the focal male, which could physically interact with a sexually mature virgin female partner. Each focal male was tested in these three situations in random order. All audience females were fertile and sexually mature. The three audience females might be recognized by the focal male as potential additional copulation partners. We presented three audience females to simulate a more natural mate-choice copying situation. Sailfin mollies live in mixed-sex shoals with a female skewed sex-ratio. Hence, it is very likely that more than one female simultaneously observes a sexual interaction between another female and a male in the wild. Virgin female partners were used only once.

#### Experimental set-up

**Figure 2:**
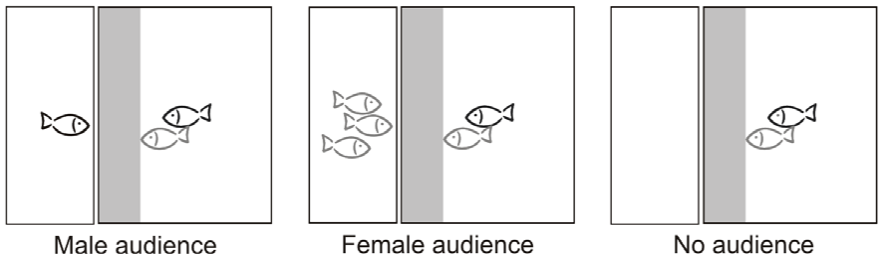
Three treatments of the sperm transfer experiment. We used a tank for the focal male and a virgin female partner, and a separate tank for a single male audience (matched for body length with the focal male, left), three fertile females (matched for body length with the female partner) as audience (middle) and no audience (right). We videotaped the experiment and measured different types of courtship behaviour and the time the focal male and the female partner spent alone and together in the 5-cm inspection zone (grey area) in front of the audience tank. Males are presented as black fishes, females as grey fishes.

We used a tank (20 cm×25 cm×41 cm) for the focal male and his female partner and an audience tank (10.5 cm×25 cm×41 cm) adjacent to the smaller side of the test tank for the audience fish (see Fig. 2). Each tank was filled with water up to 25 cm and had no gravel on the ground. Water temperature was 22±1°C. A 5 cm deep zone (inspection zone) adjacent to the audience tank was marked with a black vertical bar on the front glass of the test tank. Before starting the experiment, a removable opaque screen visually separated the two tanks. All fish could acclimatise for 10 min.

First, we gently placed a male audience, three fertile females or no audience in the audience tank and a virgin female in the test tank, while the focal male was placed in a clear Plexiglas cylinder (11 cm diameter) inside the test tank to prevent pair interaction prior to the start of the experiment. Then, we removed the screen and released the focal male out of the Plexiglas cylinder.

The focal male and his female partner could physically interact with each other for the next 30 min. When no copulation occurred within this period, the observation period was extended until first copulation (max. 60 min). All interactions were video recorded (Panasonic HDC-SD 100), and we analysed all courtship behaviours and copulations. We measured the latency until first copulation. We measured how often the male followed the female, the number of gonopodial swinging, the number of genital nipping, the number of courtship displays and copulations. We also measured the time the male and the female spent alone and together in the inspection zone.

Two - three days before the experiment, we measured the amount of stored sperm inside males and after each experiment, we measured the amount of sperm which was transferred from the male to his female partner and the amount of sperm that remained in the male (see *sperm sampling*). After each experiment, we measured the standard body length of all fish.

#### Sperm sampling

To get a ‘baseline’ of the amount of sperm for individual males and to monitor the amount of sperm of individual males during the total testing period, we isolated focal males (*N* = 15) from females for 14 days and stripped the males first time three days before the first trial and then 2.4±0.2 days before each following trial of the sperm transfer experiment. Three days are sufficient for males to recover their sperm reserves in sailfin mollies [109].

To measure the amount of sperm males transferred to females and how much sperm remained inside focal males, we gently stripped the female partner and focal male after each trial of the sperm transfer experiment. For this procedure, we used the protocol from Schlupp and Plath [107] except that we used microcentrifuge tubes (1.5 ml, Molecular BioProducts) and transferred sperm from males into 200 µl and from females into 50 µl of a 0.9 M NaCl solution.

#### Sperm count

We transferred 10 µl of the sperm containing solution to a Neubauer improved counting chamber covered with a slide, and counted the spermatozoa with a microscope (Zeiss Axiostar plus) at a 400x magnification. Spermatozoa were quantified in six randomly chosen fields, and the mean from all six fields was calculated. Spermatozoa touching the upper and right line of a field were counted for the sample; those touching the lower and left line were omitted. From the resulting data for the known volume of the counting chamber, the total number of sperm suspended in the aliquot was calculated.

#### Focal males, virgin female partners and audience fish

Virgin females (*N* = 44) had an average body length of 32.0±5.1 mm (mean ± SD).

In the treatment with an audience male, focal males (*N* = 15) and audience males (*N* = 15) were matched for body length (focal males: 46.7±9.4 mm; audience males: 47.3±9.2 mm). In the treatment with audience females, we had to remove one test from the analysis because the virgin female was aggressive to the focal male. The female attacked the male several times and prevented copulation. In this treatment, virgin females (*N* = 14) and audience females (*N* = 42) were matched for body length (body length of virgin females: 32.7±5.4 mm and audience females: 31.3±5.1 mm).

In the treatment without audience, focal males were on average 46.7±9.4 mm large and virgin females were on average 31.5±4.0 mm large. In our experiment, males were bigger than females because we used virgin females, which were at least 6 months old and were reared especially for this experiment. Males used in this experiment were up to three years old and, therefore, bigger than the virgin females. To avoid any effects due to differences in body length of virgin females (i.e. males transfer more sperm to a bigger female partner than to a smaller female partner), we used virgin females of the same body length.

### Data Analysis

Data analyses were carried out with SPSS (IBM Statistics 20). All *P*-values were two-tailed (α = 0.05), and all data were tested for normality with a Kolmogorov-Smirnov-test. Since our data were not normally distributed, we used non-parametric statistics. Since absolute association time was not normally distributed, we presented absolute association time as median plus 1. and 3. quartile. Descriptive statistics are given as mean ± SD or median with quartiles.

Because the absolute time males spent in front of females significantly decreased in the second mate-choice test of the audience experiment (Wilcoxon matched-pairs test: *Z* = −2.981, *N* = 12, *P* = 0.003), we did not compare the absolute time males spent in front of the females. Instead, we calculated a mate-choice score (time males spent with the larger or smaller female/time spent with both females) for focal males in the first mate-choice test (no audience male) and in the second mate-choice test (with an audience male). We performed an arcsine-square root transformation with the mate-choice scores and compared mate-choice scores using a rmGLM, with mate-choice scores as the dependent variable and treatment (audience present or absent) as a fixed factor and including focal male body length, audience male body length, stimulus female body length, differences in female body length as covariates, and mate-choice scores in the first mate-choice test and in the second mate-choice test as within-subject factors (repeated measurement). We did the same analysis for the control in which we tested other focal males and other stimulus females.

Differences between the treatments of the sperm transfer experiment were estimated by a Friedman test and a Bonferroni corrected Wilcoxon matched-pairs test as a Post-hoc test.

## Results

Neither in the audience experiment (F ≤0.955, P≥0.354) nor in the control (F ≤4.717, P≥0.058), did any of the covariates or any interaction term have a significant effect. Thus, we excluded these covariates from the model.

### Audience Experiment

Focal males and audience males were matched for body length (Mann-Whitney U, *N_1_* = 12, *N_2_* = 12, Z = −0.435, *P* = 0.671). Stimulus females differed significantly in standard body length (Mann-Whitney U, *N_1_* = 12, *N_2_* = 12, Z = −2.211, *P* = 0.027) in each test.

Males spent 1024.0 s (1. quartile 596.0 s, 3. quartile 1077.0 s) with larger females and only 70.0 s (1. quartile 43.0 s, 3. quartile 413.8 s) with smaller females in the first mate-choice test. In the second mate-choice test (with an audience male), focal males spent only 296.5 s (1. quartile 202.8 s, 3. quartile 413.0 s) with larger females and 243.0 s (1. quartile 111.8 s, 3. quartile 434.0 s) with smaller females.

**Figure 3:**
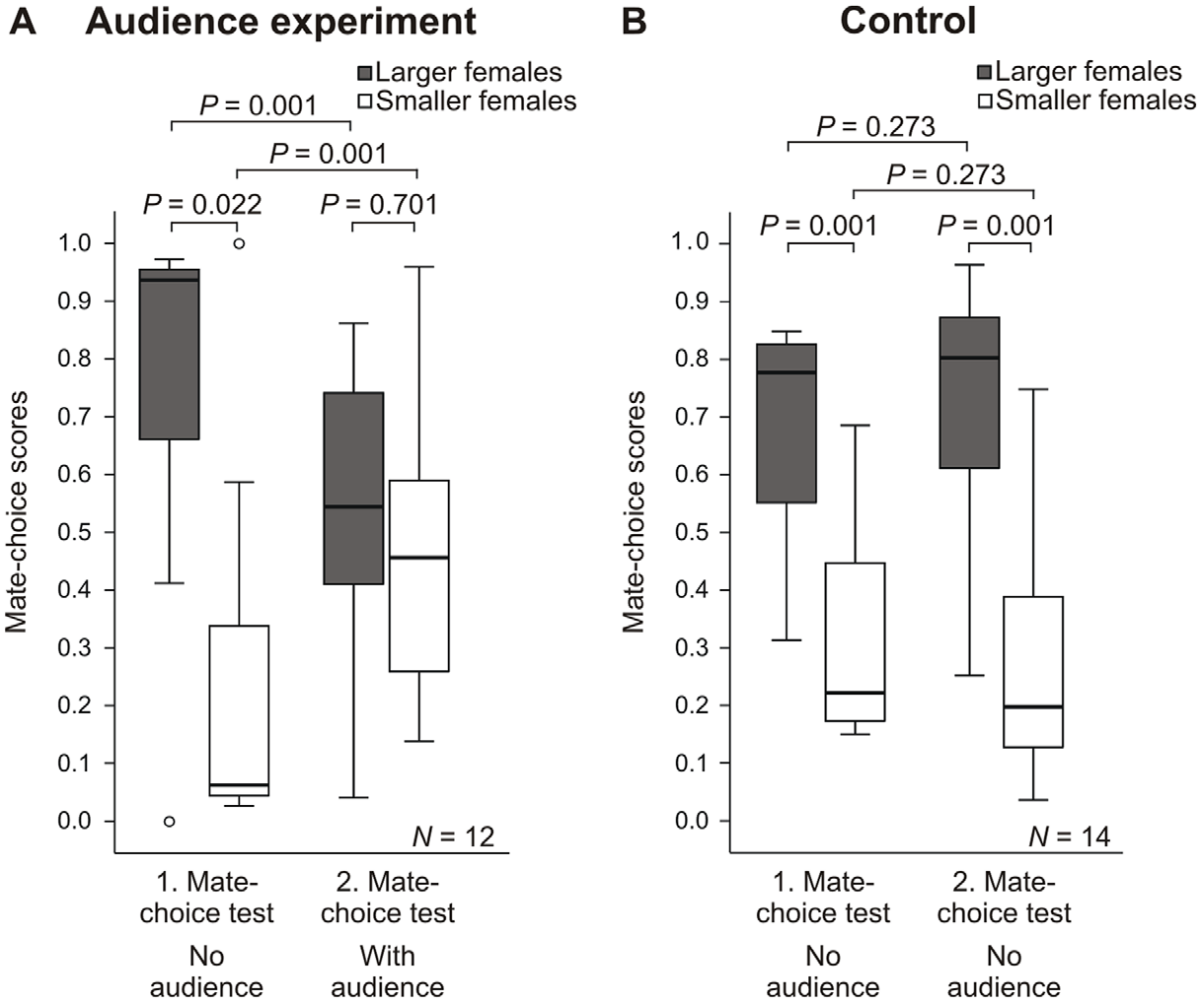
Mate-choice scores of males in the audience experiment and in the control. Mate-choice scores of males ( = time spent with the larger or smaller female/time spent with both females). In the audience experiment (A) an audience male was present in the second mate-choice test: In the control (B) no audience (empty Plexiglas cylinder) was present in the second mate-choice test.

In the first mate-choice test, focal males preferred larger over smaller females (see Fig. 3; rmGLM: df = 1, *F* = 7.167, *P = *0.022). Nine of 12 males preferred larger females. In the presence of an audience male, focal males significantly reduced time spent with larger females and significantly increased the time spent with smaller females (rmGLM: df = 1, *F* = 18.680, *P = *0.001, both), and they did not exhibit a preference for larger females in the second mate-choice test (rmGLM: df = 1, *F* = 0.155, *P = *0.701). Only 6 of 12 males preferred larger females in the second mate-choice test.

#### Control for the audience experiment

Stimulus females differed significantly in body length in each test (Mann-Whitney U: *N_1_* = *N_2_* = 13, Z = −3.057, *P* = 0.002, one data point missing).

Males spent 668.0 s (1. quartile 531.8 s, 3. quartile 799.5 s) with larger females and 207.0 s (1. quartile 154.3 s, 3. quartile 445.3 s) with smaller females in the first mate-choice test. In the second mate-choice test (with an empty Plexiglas cylinder), focal males spent 617.0 s (1. quartile 406.8 s, 3. quartile 785.3 s) with larger females and 195.0 s (1. quartile 80.8 s, 3. quartile 238.8 s) with smaller females.

In the first mate-choice test of the control, focal males preferred larger over smaller females (see Fig. 3; rmGLM: df = 1, *F* = 17.187, *P = *0.001). Ten of 14 males preferred larger females. Without an audience, there was no change in mate-choice scores for larger or smaller females in the second part (rmGLM: df = 1, *F* = 1.310, *P = *0.273, both), and males preferred larger females in the second mate-choice test, too (rmGLM: df = 1, *F* = 18.958, *P = *0.001). In the second mate-choice test, 12 of 14 males preferred larger females.

When directly comparing the second mate-choice test in the audience experiment with the second test in the control performed with other males, we found a significantly weaker strength of preference (measured as the difference of mate-choice score of larger females – mate-choice score of smaller females; Mann-Whitney U: *N = *26, *Z* = −2.160, *P* = 0.031). Thus, there was an audience effect in sailfin molly males.

### Sperm Transfer Experiment

#### Sperm sampling – baseline

During the total course of the experiment, the amount of sperm inside focal males did not decrease (in total 11.5±1.3 days). There was no significant difference over time in the amount of sperm we measured before each treatment, i.e. between treatments (Friedman test: *N = *14, df = 2, *P* = 0.395), nor during the treatments, i.e. within treatments (Friedman test: *N* = 14, df = 2, *P* = 1.0). Sperm number was highly variable between focal males. Focal males (*N = *15) contained a median of 10.34×10^5^ sperm (1. quartile 9.17×10^4^, 3. quartile 14.95×10^5^).

#### Body length of fish

Virgin females used in different treatments did not differ in body length (Kruskal Wallis: *N_1_* = 15, *N_2_* = 14, *N_3_* = 15, df = 2, *P* = 0.697).

In the treatment with an audience male, focal males and audience males were matched for body length (Mann-Whitney U: *N_1_* = 15, *N_2_* = 15, Z = −0.063, *P* = 0.950). In the treatment with audience females, virgin females and audience females were matched for body length (Mann-Whitney U: *N_1_* = 14, *N_2_* = 42, Z = −0.961, *P* = 0.336).

#### Sperm transfer

**Figure 4:**
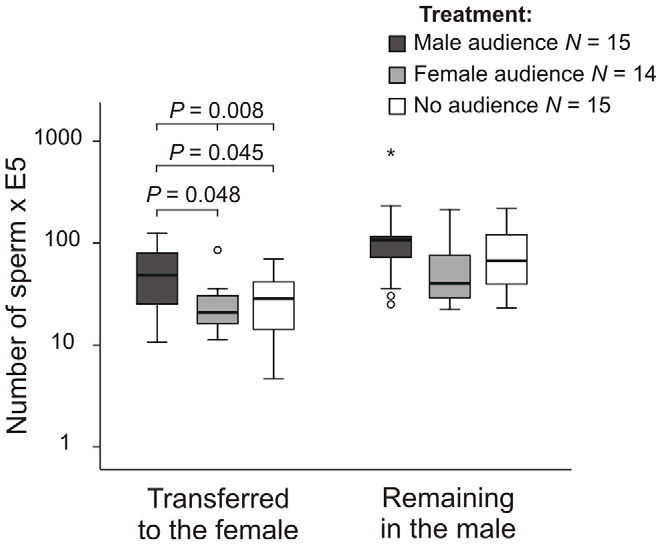
Sperm transferred to females and sperm remaining in males. Median number of sperm transferred from the male to the female partner and remaining in the male in the three treatments (*P*-values after Bonferroni correction).

Focal males transferred a significantly different amount of sperm to females in different treatments (Friedman test: *N* = 14, df = 2, *P* = 0.008). They transferred significantly more sperm to the female partner in the presence of a male audience and significantly less sperm in the presence of the three-female audience (Post hoc: Wilcoxon matched-pairs test: male audience vs. no audience, *N* = 15, Z = −2.442, *P* = 0.045; male audience vs. female audience, *N* = 14, Z = −2.417, *P* = 0.048, after Bonferroni correction, Fig. 4).

**Figure 5:**
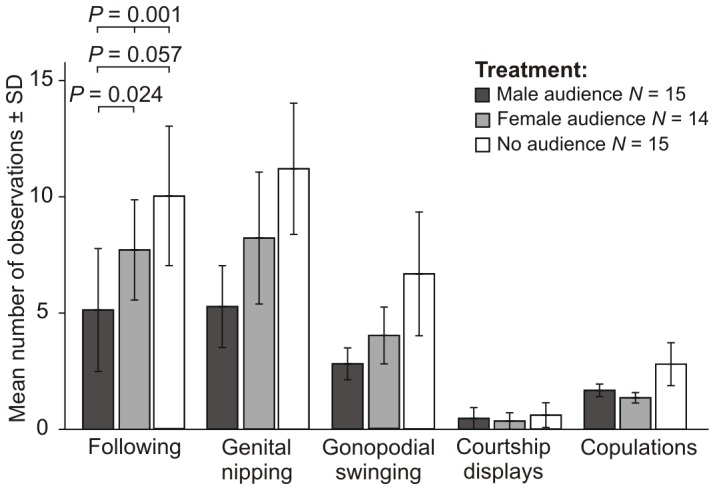
Courtship behaviour of focal males in the three treatments (*P*-values after Bonferroni correction).

Focal males also changed their mate-choice behaviour to the female partner in the three different treatments (see Fig. 5). They followed the female partner significantly less often in the presence of an audience male than in the presence of a three-female audience or no audience (Friedman test: *N* = 14, df = 2, *P* = 0.001; Post hoc: Wilcoxon matched-pairs test, male audience vs. no audience, *N* = 15, Z = −2.345, *P* = 0.019; male audience vs. female audience, *N* = 14, Z = −2.643, *P* = 0.008; after Bonferroni correction: male audience vs. no audience *P* = 0.057; male audience vs. female audience *P* = 0.024). Males performed less often genital nipping in the presence of a male audience compared to no audience or a female audience (Friedman test: *N* = 14, df = 2, *P* = 0.025; Post hoc: Wilcoxon matched-pairs test, male audience vs. no audience, *N* = 15, Z = −1.949, *P* = 0.051; male audience vs. female audience, *N* = 14, Z = −2.101, *P* = 0.036; after Bonferroni correction: male audience vs. no audience *P* = 0.153; male audience vs. female audience *P* = 0.108). All other parameters, like courtship displays, gonopodial swinging, number of copulations and time until the first copulation did not differ significantly between the treatments (Friedman test: *N* = 14, df = 2, all *P*>0.156). Our results showed that males invested less time in courting their female partners in the presence of an audience male.

**Figure 6:**
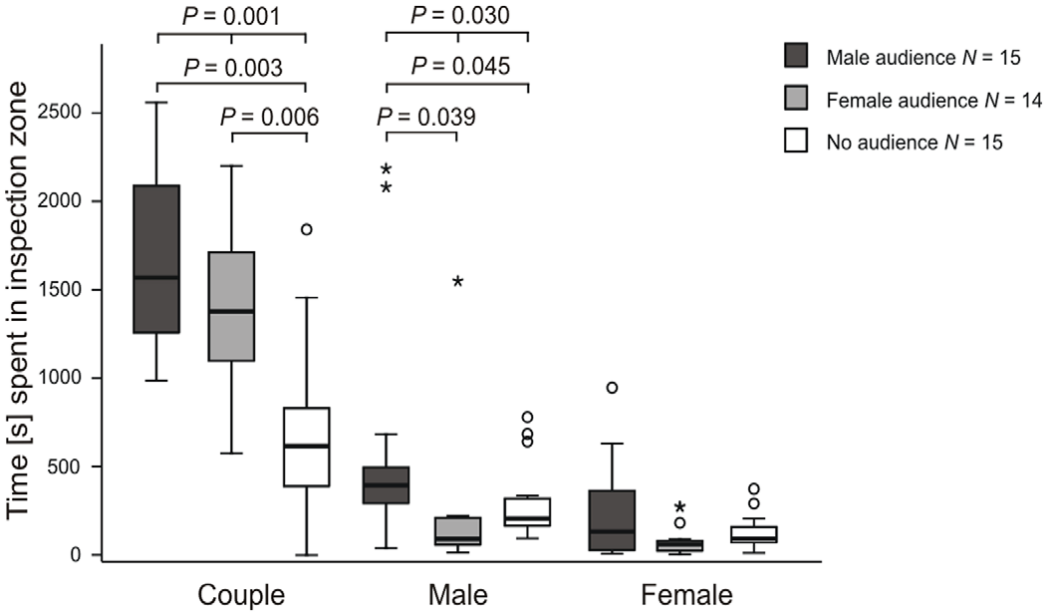
Time spent of focal males and their female partners in the inspection zone in front of the audience tank (*P* -**values after Bonferroni correction).**

The couple spent significantly more time within the 5-cm inspection zone in front of the adjacent tank (see Fig. 6; Friedman test: *N* = 14, df = 2, *P* = 0.001) when a male audience or the three-females audience was present compared to no audience (Post hoc: Wilcoxon matched-pairs test, male audience vs. no audience, *N* = 15, Z = −3.237, *P* = 0.003; female audience vs. no audience, *N* = 14, Z = −3.107, *P* = 0.006). Thus, both, focal males and female partners have recognized the audience fish. The time the focal male spent alone in the inspection zone was also influenced by the presence of an audience (Friedman test: *N* = 14, df = 2, *P* = 0.030). Focal males spent significantly more time in the inspection zone when a male audience was present compared to no audience or the female audience (Post hoc: Wilcoxon matched-pairs test, male audience vs. no audience, *N* = 15, Z = −2.442, *P* = 0.045; male audience vs. female audience, *N* = 14, Z = −2.480, *P* = 0.039). Our results showed that focal males and the female partners recognized the audience. When an audience, especially a male audience, was present they spent more time in the inspection zone.

## Discussion

In this study we could show that sailfin molly males were influenced in their mate-choice behaviour as well as in the amount of sperm they transferred to a female partner by the public information they gained.

In our first mate-choice test in the audience experiment, males significantly preferred larger females over smaller females. This general preference for larger females is well documented and known to be genetically determined (e.g. [116], [117]). Due to a positive correlation between fecundity and female body length [118], mating with larger females enhances male reproductive success.

In the presence of a male audience, however, focal males spent significantly less time with the previously preferred larger of two females and significantly more time with the previously non-preferred smaller female. In the control, without an audience, males were highly consistent in their mate choice and even significantly increased time spent with the preferred larger female in the second mate-choice test. The strength of preference for larger females was significantly weaker during the audience experiment, part two, than in the control without an audience, part two. This indicates an audience effect in sailfin molly males.

Our results are in line with results from similar male mate-choice experiments in the closely related Atlantic molly, *P. mexicana* [96], [97]. In these experiments, Atlantic molly males reduced time spent in front of the initially preferred larger female. Males even preferred smaller females over larger ones in presence of an audience male [96], [97]. It was suggested that males tried to deceive their competitors with the pretended mate choice to reduce sperm competition [95]–[98]. Due to the fact that males copy the mate choice of other males, sending deceptive signals and leading competitors away from a preferred female might be an alternative mating strategy to lead other males to copy a wrong mate-choice decision [75], [78], [95], [97].

The most important question, however, is how the use of public information is linked to fitness. One mechanism might be that males adjust sperm transfer to a female partner according to public information, i.e. to the specific type of audience. We investigated this question in the second experiment, the sperm transfer experiment. Although we performed two separated experiments, both experiments were strongly linked to each other. In the audience experiment, we found a change in male mate-choice behaviour due to the presence of a male audience, and in the sperm transfer experiment, we investigated whether males were able to adjust sperm transfer to a female partner in the presence of a male audience.

In the sperm transfer experiment, we could show that public information, i.e. the type of an audience, affected fitness investment in sailfin mollies measured as the amount of sperm transferred to a female when males could physically interact with a female and were faced with different types of audience. Males transferred significantly more sperm to a female partner in the presence of an audience male than in the presence of a female audience or no audience. In the presence of a male audience, focal males spent less time courting their female partner. Thus, males seem to be more efficient in transferring more sperm to a female partner while spending less time courting.

Sperm competition theory predicts that males should be able to adjust sperm production to the number of rival males and thus, to the level of sperm competition risk (SCR) and sperm competition intensity [119]. Parkers SCR model predicts that if there is a low probability that the males’ ejaculates will have to compete, individual males should invest less in each mating; whereas, if the probability of competition is high, males should invest more (increase ejaculate size) in each mating [119]. In the presence of competitors, males both primed more sperm prior to mating and transferred more sperm than males in the absence of a male competitor [120].

Sperm competition in natural poeciliid populations can be intense (e.g. [120]–[122]), especially in populations in which females mate multiply (e.g. [118], [123], [124]). Up to now, nine different populations of *P. latipinna* were tested and the level of multiple sired broods ranged from 9−85% [118], [123], [124]. Thus, according to sperm competition theory and public information use in mate choice, a male should transfer more sperm to the female partner in the presence of a male audience than without an audience. Our results supported this hypothesis.

Males did not only transfer significantly more sperm to their female partner in the presence of a male audience, they also reduced their courtship displays to the female partner, probably to mask their real mate choice. Probably, one strategy to mask the real mate choice is to reduce courtship displays to the female partner. The reduction of courtship display might be simply caused by a reduced attention of the focal male, since more individuals are present to interact with. If that is true, we would expect that males reduce the courtship display to the female partner most of all in the experimental treatment with three audience females. However, we found the strongest reduction in courtship in the treatment with one audience male. Thus, we suggest that the sex of the audience has a stronger influence on sperm transfer than the number of audience fish to interact with. We found a similar strategy in sailfin molly males in our audience experiment and in the sperm transfer experiment. Plath et al. [97] found that *P. mexicana* males reduced mate-choice behaviour when they could physically interact with females in presence of a male audience, whereas Makowicz et al. [100] found an increase in mate-choice behaviour in sailfin molly males when a virtual audience was presented. The focus of the study by Makowicz et al. [100] was to investigate the reduction in female feeding time due to male harassment in the sailfin molly. They tested whether a video male audience, larger or smaller than the focal male, influenced mating behaviour of the focal male. Audience males were presented on videos to prevent direct interaction between focal males and audience males. Makowicz et al. [100] predicted a decrease in male sexual behaviour to the focal female with a stronger effect on focal males when the video audience male was smaller. In contrast to their prediction, they found an increase in mean sexual activity when a video audience was present, with a stronger increase by presenting a larger audience male. In our study, we used a real audience, who could interact with the focal male. Furthermore, we used size matched audience and focal males. Although both experiments were a no-choice situation, our experiment differed in an important parameter, the live audience male. In our study, focal males spent less time courting a female partner, however, they transferred more sperm to the female partner in that situation, which is the more crucial factor for fitness than courtship activity. Our observations of reduced mate-choice behaviour and a higher amount of sperm transferred to a female in presence of a male audience are in line with the prediction that males should mask their real mate choice in the audience experiment and transfer more sperm to females to reduce sperm competition, because a male competitor is likely to show the same mate choice (e.g. for larger body size, [118]) and it is very likely to copy the focal males’ choice [75], [78]. This seems to be a good strategy to increase the likelihood of paternity in a species with multiple paternity but without a clear second male (P_2_) mating advantage [120], [125].

In the case of the female audience, males should save sperm and transfer less to the female partner to be able to copulate with the audience females immediately.

Audience females might be recognized by the focal male as potential copulating partners, which copy the choice of his actual female partner. Thus, it would be advantageous for the male to save sperm to copulate with audience females, which will accept the male as a mate due to the observed sexual interaction between the focal male and his female partner right before. Our results showed that indeed males transferred significantly less sperm to his female partner when female audience was present than when a male audience was present.

There was no significant difference in the total amount of sperm extracted in the different treatments. It seems as if the audience male has an influence on males’ priming response, which was, up to now, only known for bystanding females [126], [127]. The priming response is probably a mechanism by which males conserve energy associated with sperm production [128]. In guppies (*P. reticulata*), the availability of females significantly increases the amount of sperm that males produce for transfer. It was suggested, that the priming response helps male guppies to conserve energy resources due to sperm production, depending on the availability of females [127]. A study by Aspbury [120] could also demonstrate that male sailfin mollies primed more sperm prior to mating when exposed to males for 1 to 7 days and expended more sperm during mating in accordance with predictions of the sperm competition risk (SCR) model of Parker et al. [119]. Parkers SCR model predicts that if there is low probability that the males’ ejaculates will compete, individual males should invest less in each mating, whereas if probability of competition is high, males should invest more (increase ejaculate size) in each mating [119]. In the presence of competitors sailfin molly males both primed more sperm prior to mating and expended more sperm than males in the absence of a male competitor [120]. The model of Parker et al. [129] predicts sperm expenditure to be at the maximum when only one competing male is present. This is in line with our findings that males invested less time in courtship to mask their preference but transferred more sperm to their female partners in presence of an audience male.

Beside the fact that males adjusted the amount of sperm they transferred according to the type of audience, they also adjusted their courtship displays. In the presence of an audience male, males reduced sexual behaviour in general. They spent more time in the inspection zone when an audience male was present. Thus, focal males inspected the other male, a potential competitor. Additionally, we observed that in some cases the focal male chased the female partner out of the inspection zone, but only when an audience male was present.

In the treatment with audience females, males did not change courtship behaviour in comparison to the treatment without an audience.

Both, the audience experiment and the sperm transfer experiment showed how public information influences male mate-choice behaviour on two different levels. In the presence of a male audience, sailfin molly males changed their mat-choice behaviour, i.e. spent less time with previously preferred larger females and more time with previously non-preferred smaller females, and thus changed the probability to mate with a larger or smaller female [130]. Moreover, males adjusted the amount of sperm transferred to a female partner, a crucial measurement of fitness investment, to the presence or absence of an audience male.

To our knowledge this is the first study showing how the use of public information is not only linked to male mate-choice behaviour but also to male fitness investment. This shows further how the use of public information can influence the reproductive success of males in a population, and more generally, how the use of public information, a non-genetic factor in sexual selection, is turned into fitness, which is directly under sexual selection. Public information use, therefore, is a meaningful non-genetic factor in the dynamic processes in sexual selection.
